# Analysis on heterogeneity of hepatocellular carcinoma immune cells and a molecular risk model by integration of scRNA-seq and bulk RNA-seq

**DOI:** 10.3389/fimmu.2022.1012303

**Published:** 2022-10-13

**Authors:** Xiaorui Liu, Jingjing Li, Qingxiang Wang, Lu Bai, Jiyuan Xing, Xiaobo Hu, Shuang Li, Qinggang Li

**Affiliations:** ^1^ Department of Infection, The First Affiliated Hospital of Zhengzhou University, Zhengzhou, China; ^2^ Department of physical examination&Blood collection Xuchang Blood Center, Xuchang, China; ^3^ Bioinformatics R&D Department, Hangzhou Mugu Technology Co., Ltd, Hangzhou, China

**Keywords:** hcc, ScRNA-seq, riskscore, autophagy, molecular subtypes

## Abstract

**Background:**

Studies have shown that hepatocellular carcinoma (HCC) heterogeneity is a main cause leading to failure of treatment. Technology of single-cell sequencing (scRNA) could more accurately reveal the essential characteristics of tumor genetics.

**Methods:**

From the Gene Expression Omnibus (GEO) database, HCC scRNA-seq data were extracted. The FindCluster function was applied to analyze cell clusters. Autophagy-related genes were acquired from the MSigDB database. The ConsensusClusterPlus package was used to identify molecular subtypes. A prognostic risk model was built with the Least Absolute Shrinkage and Selection Operator (LASSO)–Cox algorithm. A nomogram including a prognostic risk model and multiple clinicopathological factors was constructed.

**Results:**

Eleven cell clusters labeled as various cell types by immune cell markers were obtained from the combined scRNA-seq GSE149614 dataset. ssGSEA revealed that autophagy-related pathways were more enriched in malignant tumors. Two autophagy-related clusters (C1 and C2) were identified, in which C1 predicted a better survival, enhanced immune infiltration, and a higher immunotherapy response. LASSO–Cox regression established an eight-gene signature. Next, the HCCDB18, GSA14520, and GSE76427 datasets confirmed a strong risk prediction ability of the signature. Moreover, the low-risk group had enhanced immune infiltration and higher immunotherapy response. A nomogram which consisted of RiskScore and clinical features had better prediction ability.

**Conclusion:**

To precisely assess the prognostic risk, an eight-gene prognostic stratification signature was developed based on the heterogeneity of HCC immune cells.

## Introduction

Primary liver cancer is a malignancy with a high degree of histological and biological heterogeneity and has therefore become a major public health problem (1). According to the Global Cancer Statistics Report, liver hepatocellular carcinoma (LIHC) was the sixth highest cancer worldwide in 2020, and its mortality rose to the third highest, accounting for 4.7% of all cancer cases and 830,000 deaths and 8.3% of all cancer deaths in the same period (2). In China, primary LIHC is the fourth most common malignant tumor and its mortality rate ranks the second in China due to historical factors, population region, and health conditions (3). Therefore, there is a strong clinical need for more effective strategies such as developing new therapeutic targets, biomarkers, and therapies for treating HCC, which remain key unmet needs for the treatment of hepatocellular cell carcinoma (HCC).

Tumor heterogeneity can be manifested as different pathological types, tumor stages, and differentiation degrees in clinical practice as well as different genomes and transcriptomes at the molecular level, which eventually lead to different sensitivities to chemotherapy and therapeutic drugs, thereby bringing great difficulties to cancer treatment (4, 5). In 2011, Navin et al. used mononuclear whole-genome amplification technology to amplify and sequence a total of 200 single nuclei in two cases of breast cancer *in situ* tissues and one case of liver metastasis tissues and analyzed their copy number changes to explain the population structure and evolution process of tumor cells (6). Patel et al. isolated 430 single cells from five brain tumor patients, conducted transcriptome analysis, and found that the internal changes in gene expression during different transcription processes were related to oncogene signaling, proliferation, immune response, and hypoxia (7). Dalerba et al. analyzed the gene expression of single cells in colonic epithelial carcinoma *in situ* tissues and normal colon tissues and observed that colon cancer tissues contained different subsets of cells and their transcripts were different from those in normal colon tissues (8).

Autophagy is a conserved lysosome-dependent pathway that degrades organelles, macromolecules, and cytoplasmic proteins in a dynamic, multistep process. Evidence suggests that autophagy has a role in tumor suppression in HCC (9, 10). For example, systemic Atg5-null mice and liver-specific Atg7−/− mice would develop benign hepatic adenomas (11), but Beclin-1 haploid deficiency could induce spontaneous HCC formation (12).

Based on the above analyses, we identified the immune heterogeneity of HCC from public databases applying bioinformatics methods and screened autophagy-related genes associated with the prognosis of HCC. Furthermore, the classification and prognostic signature of the autophagy-related genes in HCC samples were analyzed to further supplement the prognostic markers of HCC and provide new insights for clinical targeted drug therapy.

## Material and methods

### HCC data of public databases

A sum of 360 HCCs in TCGA dataset (TCGA-LIHC), 242 samples in the GSE14520 dataset, 10 HCCs in the single-cell sequencing database (GSE149614), and 389 samples in the HCCDB18 dataset were acquired from The Cancer Genome Atlas (TCGA) (13), Gene Expression Omnibus (GEO) (14), and Hepatocellular Carcinoma Database (HCCDB) (15).

Autophagy-related genes were obtained from the MSigDB (https://www.gsea-msigdb.org/gsea/index.jsp) database.

### Data control

The Seurat package (16) was used to set the expression of each gene in at least three cells (each cell expressing at least 250 genes) to filter a single cell. The proportion of rRNA and mitochondria was further calculated by PercentageFeatureSet function, and genes expressed in each cell were more than 200 but fewer than 6,000, the percentage of mitochondria was fewer than 25%, and the UMI of each cell was at least greater than 1,000. To screen highly variable genes, the FindVariableFeatures function was used, followed by scaling and PCA dimensionality reduction for all genes using the ScaleData function.

### Cell type annotation

The cells were clustered using FindNeighbor and FindCluster (Dim = 20, Resolution = 0.1). The FindAllMarker function was conducted to select marker genes. Kyoto Encyclopedia of Genes and Genomes (KEGG) pathway annotation was performed using R Package ClusterProfiler (17).

### Analysis of autophagy-related pathways

Autophagy-related pathways were obtained from GSEA (http://www.gsea-msigdb.org/gsea/index.jsp) and analyzed using the ssGSEA of GSVA package (18).

### Clustering

According to the standard of p< 0.05, oxidative stress-related genes with prognosis of HCC were filtered *via* univariate Cox survival analysis using coxph function of the R package. Then, molecular subtypes were performed on each TCGA-LIHC dataset sample *via* the ConsensusClusterPlus 1.52.0 (19). Pam arithmetic and “spearman” distance were utilized to complete 500 bootstraps with every bootstrap containing ≥80% of TCGA-LIHC dataset specimens. Cluster number k was between 2 and 10, and the optimum k was identified as per cumulative distribution function (CDF) and AUC. Survival curves (K–M curves) between molecular subtypes were then analyzed for difference. In addition, differences in the distribution of clinical characteristics between molecular subtypes were compared and the chi-square test was conducted, and p*<*0.05 indicated statistical significance.

### Mutation analysis

A waterfall plot was generated to explore the detailed single-nucleotide variant (SNV) characteristics between molecular subtypes *via* the “mutect2” (20) function in R software.

### Construction and evaluation of a prognostic risk model for HCC

LASSO–Cox regression was conducted in the glmnet package in R language to select prognostic genes (21). By penalized maximum likelihood, glmnet fits generalized linear and similarity models. The regularization path is the calculation of the LASSO or elastic net penalty on the value (on a logarithmic scale) of the regularization parameter lambda (22). The genes included in the model and the optimal value of the penalty coefficient λ were determined through running a 1,000-time 10-fold cross-validation probability. Subsequently, coefficients of prognostic genes were extracted by Cox multivariate regression analysis, and the gene expression levels were used to calculate the risk score as the survival risk score of each patient by the following formula:


$$RiskScore=\sum_{k=0}^{n}\beta i\times Expi$$

where βi is the Cox hazard ratio coefficient of mRNA and Expi represents the gene expression level. TCGA-LIHC samples were divided into high-risk and low-risk groups according to the risk score, with the median risk score as the threshold. At the same time, GSE14520 and HCCDB18 were used to analyze the robustness and effectiveness of the prognostic risk model. Kaplan–Meier (K–M) curves combined with the log-rank test were applied to analyze survival differences among different risk groups. The timeROC package was employed to determine the area under the receiver operating characteristic curve (AUC) to predict the 5-, 4-, 3-, 2-, and 1-year survival rates, respectively.

### Nomogram

To further evaluate the predictive efficacy of the risk score model, a nomogram was constructed by combining other clinicopathological characteristics of HCC patients (including family history, TNM stage, age, histological grade, gender, etc.) using the RMS R package (23). The predictive accuracy of the nomogram was assessed by calculating the C-index, which quantifies the degree of agreement between the actual and predicted survival rates. The abscissa was the 1-, 3-, and 5-year survival probability of each patient predicted according to the nomogram, and the ordinate was the actual 1-, 3-, and 5-year survival probability of each patient, with the 45° line representing the optimal prediction.

### Cell-type identification using estimating relative subsets of RNA transcripts

Cell-type Identification using Estimating Relative Subsets of RNA Transcripts (CIBERSORT) analyses were utilized to compare diversities in different immunocytes in molecular subtypes. Wilcox test analyses were completed to identify the difference of 22 kinds of infiltrating immunocyte score among molecular subtypes. The “ggplot2” package (24) was used to visualize the distributional status of the diversities in 22 kinds of infiltration immunocytes.

### Estimate

The R software Estimation of Stromal and Immune cells in Malignant Tumors using Expression data (ESTIMATE) arithmetic (25) was utilized to compute overall stroma level (StromalScore), immunocyte infiltration (ImmuneScore), and combination (ESTIMATEScore) of samples in the TCGA-LIHC cohort using Wilcox test analysis for analyzing the differences between molecular subtypes.

### Tumor immune dysfunction and exclusion

The Tumor Immune Dysfunction and Exclusion (TIDE) (26, 27) algorithm (http://tide.dfci.harvard.edu) evaluated three cell types that limit T-cell invasion into tumors, including IFNG, myeloid suppressor cells (MDSC), and M2 subtypes of tumor-associated macrophages (TAM.M2), dysfunction of tumor infiltration cytotoxic T lymphocytes (CTL) (Dysfunction), and exclusion of CTL by immunosuppressive factors (Exclusion).

### Drug sensitivity analysis

pRRophetic (28) was used to predict the sensitivity of erlotinib, sunitinib, paclitaxel, VX-680, TAE684, and crizotinib to IC50. We used Sangerbox for assisting data analysis (29).

## Results

### Definition of cell clusters and dimensionality reduction

The flowchart of this work is shown in **Figure S1**. The “Seurat” function and PercentageFeatureSet function were performed to screen 33,117 cells from the scRNA-seq dataset. The UMI was obviously correlated with numbers of mRNAs (**Figure S2A**). **Figures S2B, C** display the samples before and after quality control. PCA revealed that most HCC patients were distributed by cluster (**Figure S2D**). We conducted the “ScaleData” function to scale all genes extracted from the scRNA-seq dataset (GSE149614) and performed PCA dimensionality reduction to find anchor points. Finally, 11 clusters were found based on FindNeighbors and FindClusters functions.

Cell annotation of 11 clusters was performed in terms of classical markers of immune cells. Macrophage (cluster 1; markers: CD163, CD68, CD14), T cells (clusters 2, 8; markers: CD2, CD3D, CD3E, and CD3G), B cells (cluster 9; markers: CD19, CD79A, and MS4A1), plasma cells (cluster 4; markers: CD79A and JSRP1), mast cells (cluster 10; markers: TPSAB1 and CPA3), fibroblasts (cluster 7; markers: ACTA2, PDGFRB, and NOTCH3), endothelial cells (cluster 6; markers: PECAM1), and hepatocellular carcinomas (cluster 0, 3, 5; GPC3, CD24, and MDK) were clustered according to immune cell markers (**Figure S3**).

**Figure 1:**
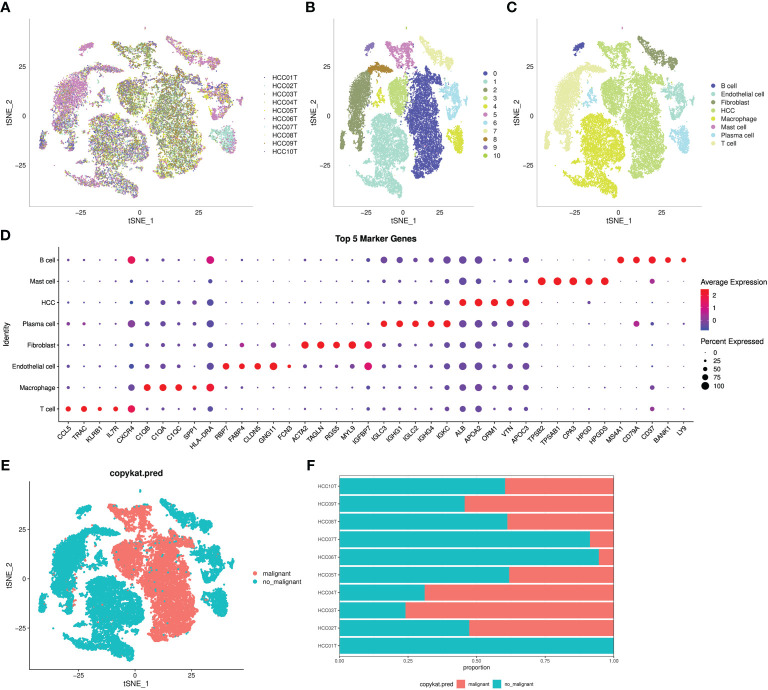
Definition of cell clusters. **(A)** t-SNE of 10 samples in the GSE149614 dataset. **(B)** t-SNE of 11 cell subgroups. **(C)** t-SNE of eight cell types. **(D)** Top five genes that made the most significant contribution. **(E)** t-SNE of malignant and no_maliganant. **(F)** The proportion of malignant and no_maliganant in 10 samples in the GSE149614 dataset.

An overview of the single cells from 10 samples in the GSE149614 dataset are listed in **Figure 1A**. All the cells were classified into 11 clusters (**Figure 1B**). Eleven clusters were labeled as eight cell types by immune cell marker genes (**Figure 1C**). The top five genes with the most prominent contributions are shown in **Figure 1D**. Next, the CNV of eight cell types was predicted using the CopyCat package to identify 13,617 malignant (tumor) cells and 19,500 no_malignant (normal) cells (**Figure 1E**). Moreover, we calculated the proportion of malignant and no_malignant cells in 10 samples (**Figure 1F**).

### Analysis of autophagy pathways

**Figure 2:**
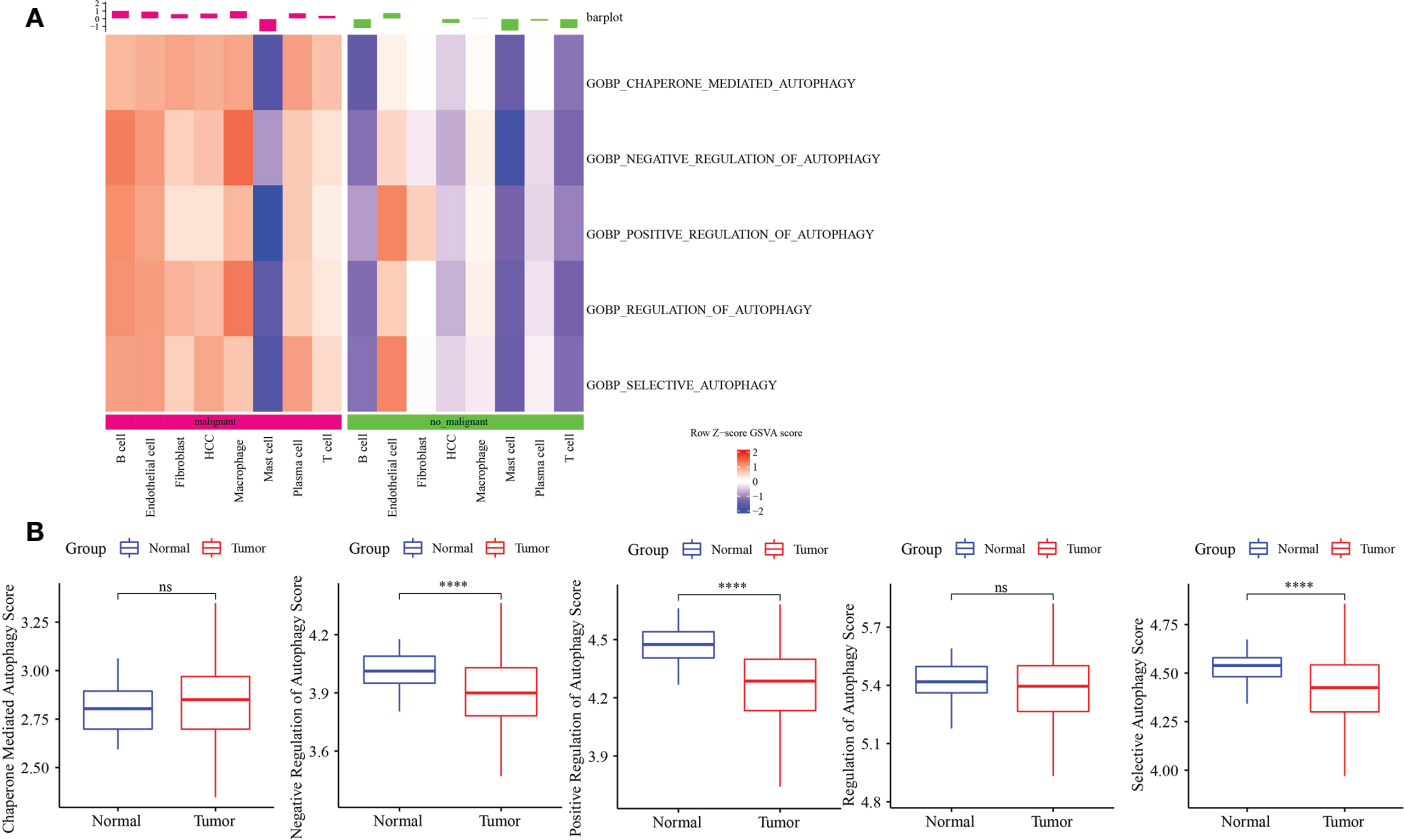
Autophagy-related pathway analysis. **(A)** Five autophagy-related pathways were activated in malignant. **(B)** The difference analysis of autophagy-related pathways scores in normal and tumor. ****p<0.0001, ns, no significance.

ssGSEA showed that autophagy pathways were activated in malignant tumors (**Figure 2A**). Next, ssGSEA of five autophagy pathways in TCGA-LIHC dataset showed that three autophagy pathways scored lower in tumors compared to the normal ones (**Figure 2B**), which was the opposite as shown in **Figure 2A**. Thus, we further analyzed the autophagy pathway scores in grade 1 to grade 4 (**Figure S4**). Here, with the increase in tumor grade, autophagy pathway scores were continuously decreased, indicating that the body was abnormal and the cells had autophagy; however, as the disease deteriorated, autophagy was gradually weakened.

### Identification of autophagy-related clusters

**Figure 3:**
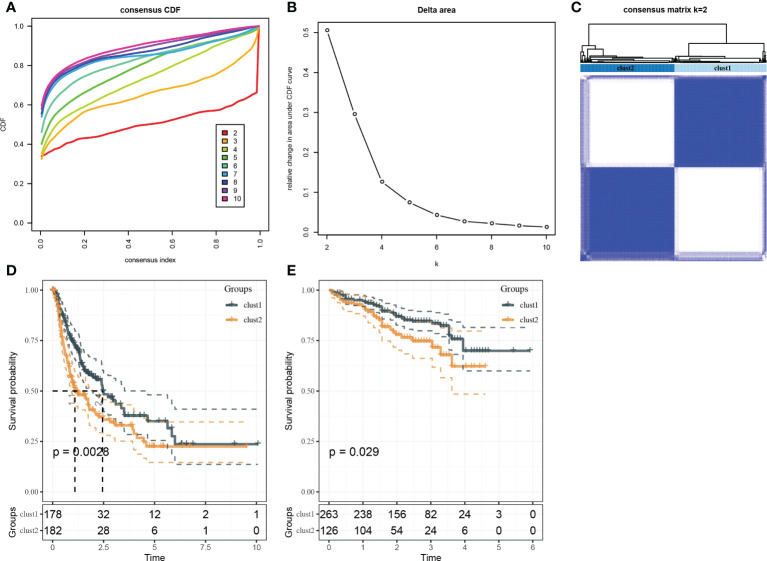
Identification of molecular subtypes. **(A)** Cumulative distribution function. **(B)** Delta area. **(C)** Heatmap of sample clustering when k = 2. **(D)** K–M survival analysis of Clust1 and Clust2 in TCGA-LIHC dataset. **(E)** K–M survival analysis of Clust1 and Clust2 in the HCCDB18 dataset.

**Figure 4:**
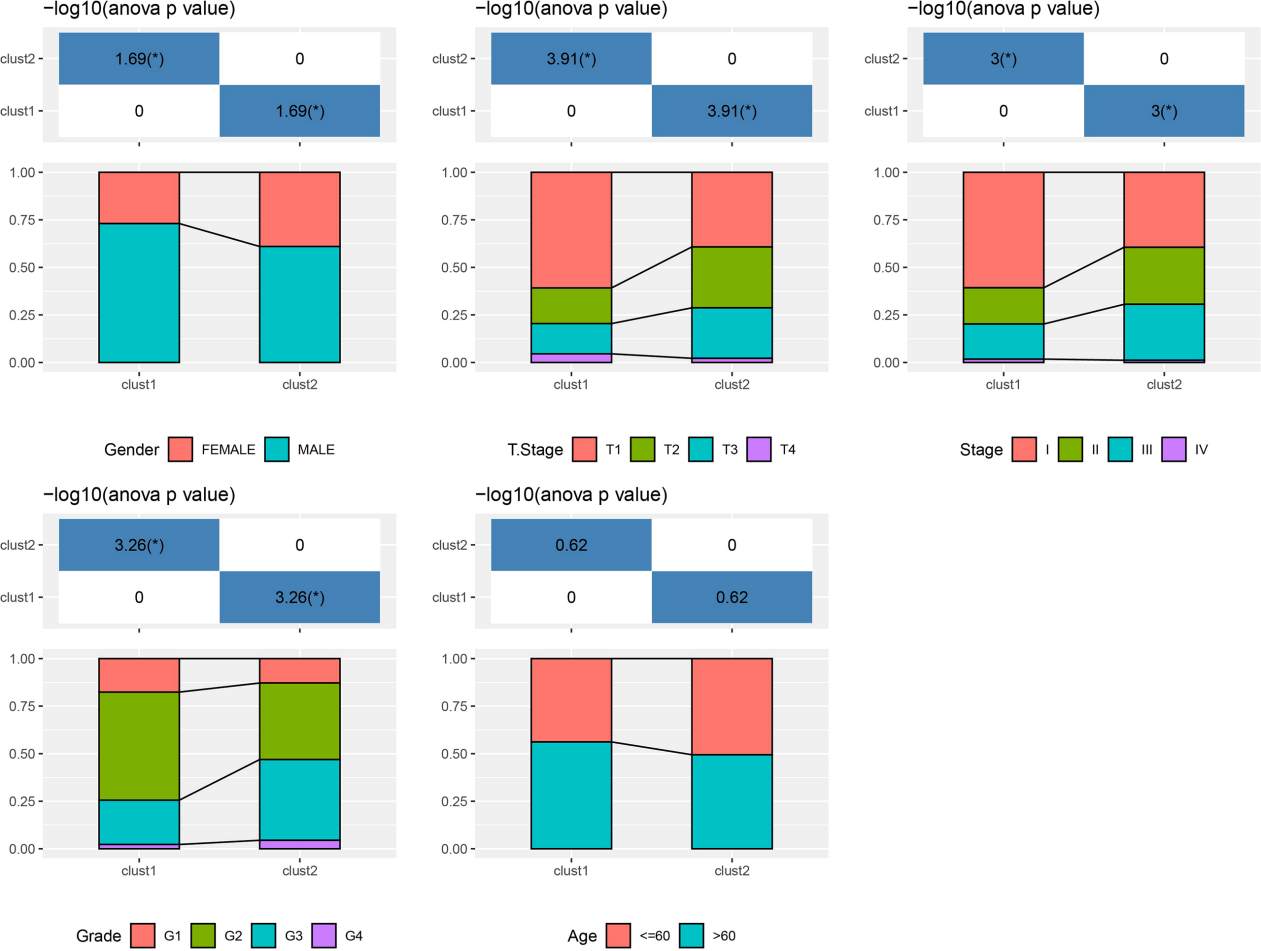
The distribution of clinical features, including gender, T stage, stage, grade and age, in Clust1 and Clust2.

Based on the results in **Figure 2B**, 253 genes in the intersection of TCGA-LIHC dataset and three autophagy pathways were analyzed by limma to obtain 38 differentially expressed genes. Three hundred sixty samples in TCGA-LIHC were clustered using the ConsensusClusterPlus package based on 38 genes. According to cumulative distribution function (CDF) and delta area (**Figures 3A, B**), two clusters (Clust1 and Clust2) were obtained when k = 2 (**Figure 3C**). K–M analysis demonstrated that HCC patients in Clust2 tended to have a shorter survival than those in Clust1 in TCGA dataset (**Figure 3D**) and HCCDB18 dataset (**Figure 3E**). Clust2 had more women, a higher T stage and clinical stage, and a poorer tumor grade (**Figure 4**).

### Genome analysis and functional enrichment analysis

**Figure 5:**
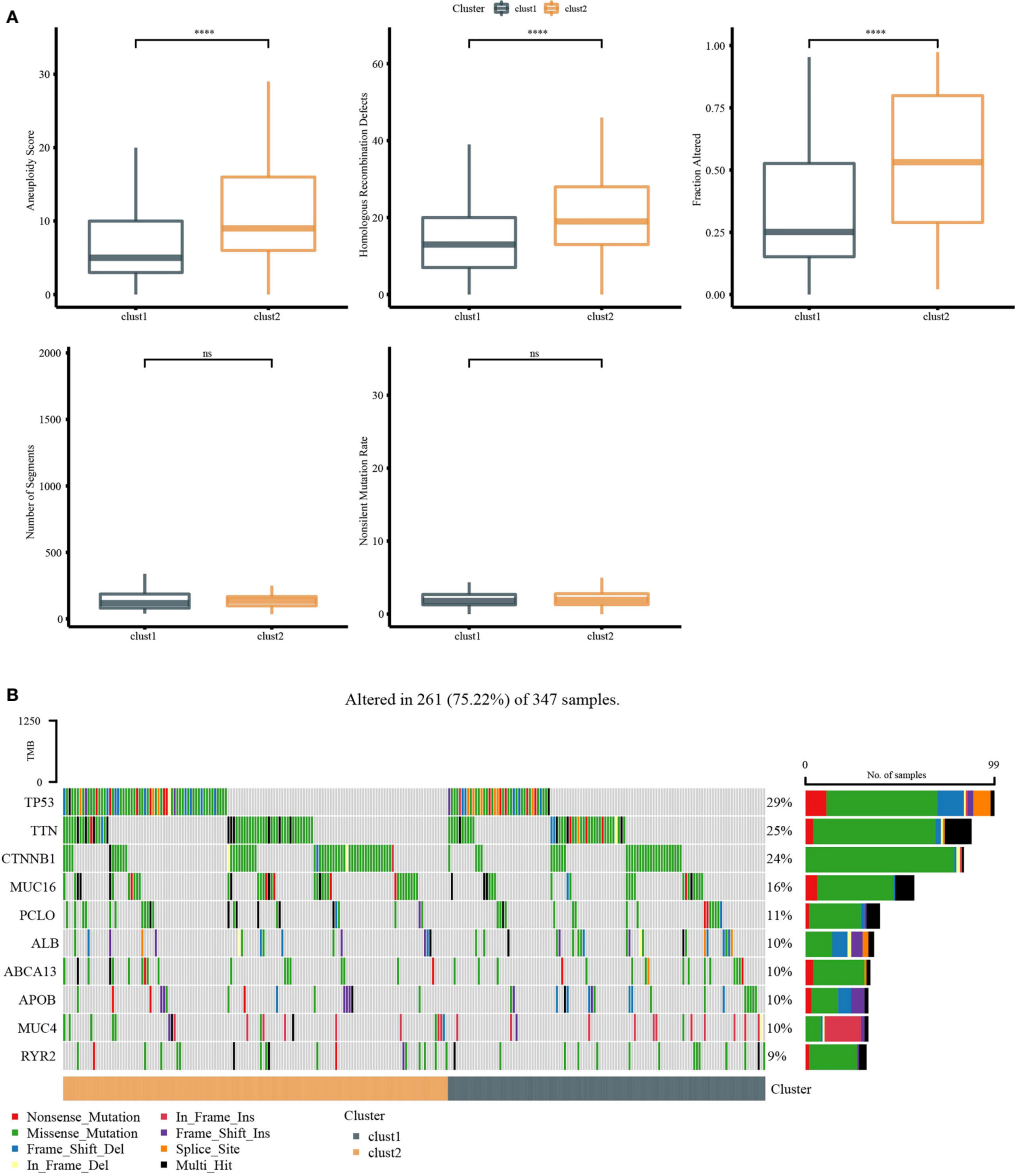
Genome analysis. **(A)** The analysis of aneuploidy score, homologous recombination defects, fraction altered, number of segments, and non-silent mutation rate in Clust1 and Clust2. **(B)** Top 15 mutation genes in Clust1 and Clust2. ****p<0.0001, ns, no significance.

**Figure 6:**
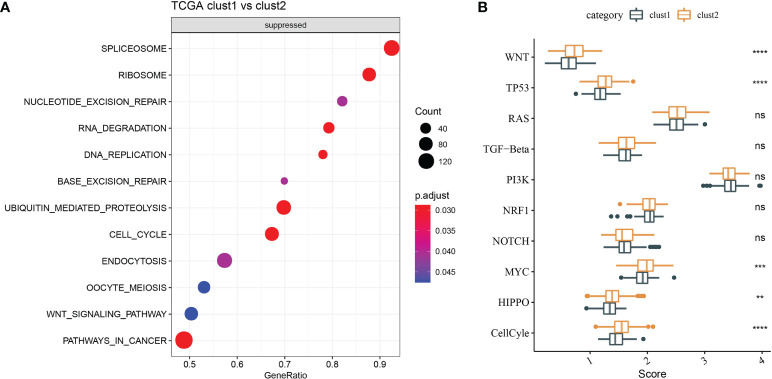
Functional enrichment analysis. **(A)** GSEA demonstrated that pathways, such as cell cycle, were activated in Clust1. **(B)** Five of 10 pathways associated with tumorigenesis had higher scores in Clust2. **p<0.01, ***p<0.001, ****p<0.0001, ns, no significance.

Molecular characteristics of TCGA-LIHC were obtained from a previous study (30). Clust1 presented a lower aneuploidy score, homologous recombination defects, and fraction altered (**Figure 5A**). The differences in gene mutation between clusters were analyzed; the top 10 genes are shown in **Figure 5B**. TP53 and TTN had obvious differences between two clusters (**Figure 5B**). GSEA showed that pathways such as cell cycle were activated in Clust1 (**Figure 6A**), and five of 10 pathways associated with tumorigenesis had higher scores in Clust2 (**Figure 6B**).

### Analysis of the tumor immune microenvironment

**Figure 7:**
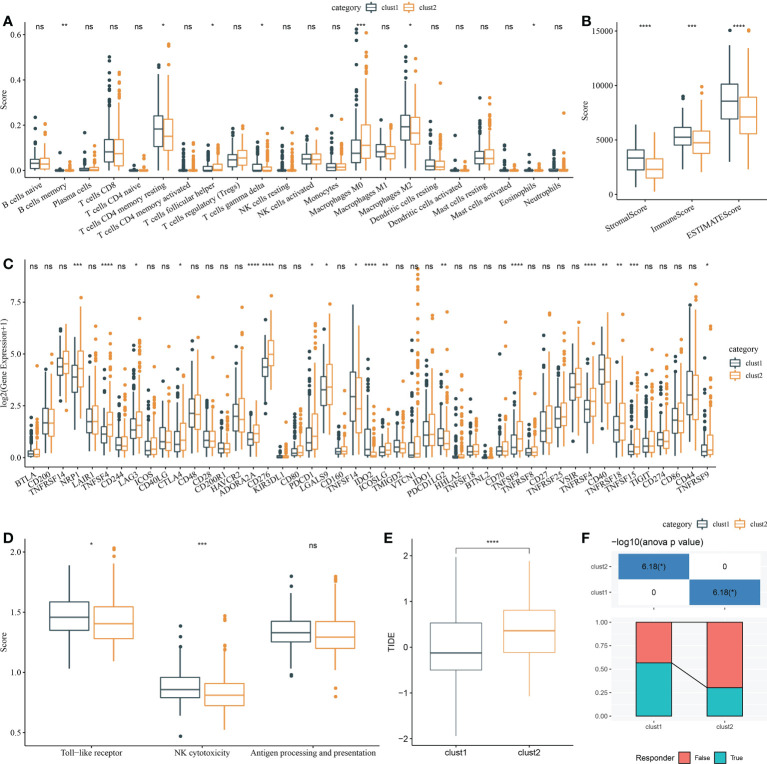
Analysis of immune infiltration. **(A)** Analysis of 22 immune cells using CIBERSORT. **(B)** Analysis of immune infiltration using ESTIMATE. **(C)** The expression levels of 42 immune check genes between Clust1 and Clust2. **(D)** The differences in Toll-like receptor signaling pathway score, natural killer cell-mediated cytotoxicity score, and antigen processing and presentation score between Clust1 and Clust2. **(E)** The differences in TIDE between Clust1 and Clust2. **(F)** Responses to immunotherapy between Clust1 and Clust2. *p<0.05, **p<0.01, ***p<0.001, ****p<0.0001, ns, no significance.

Firstly, CIBERSORT analysis indicated that seven of 22 immune cells had a significant difference between two clusters (**Figure 7A**). Then, ESTIMATE analysis showed that Clust1 had higher scores of StromalScore, ImmuneScore, and ESTIMATEScore (**Figure 7B**). We then evaluated the 47 immune check gene expressions, and 18 immune checkpoint genes had obviously high expressions in Clust2 than those in Clust1 (**Figure 7C**). Next, the scores of Toll-like receptor signaling pathway, natural killer cell-mediated cytotoxicity, antigen processing, and presentation were calculated using ssGSEA, and here it has been observed that the Toll-like receptor score and NK cytotoxicity score were higher in Clust1 than those in Clust2 (**Figure 7D**). TIDE was lower in Clust1 than in Clust2 (**Figure 7E**), suggesting that patients in Clust1 were more likely to benefit from immunotherapy and more patients in Clust1 responded to immunotherapy (**Figure 7F**).

### Identification of hub autophagy related genes and RiskScore

**Figure 8:**
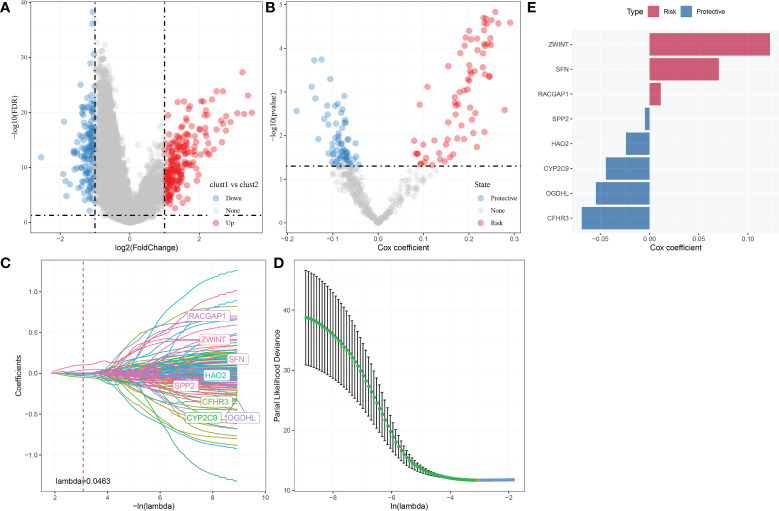
Identification hub autophagy-related genes. **(A)** Volcano plot of differentially expressed genes identified from autophagy-related pathways between Clust1 *vs*. Clust2. **(B)** Volcano plot of differentially expressed genes identified using univariate Cox analysis. **(C)** Lambda trajectory of differentially expressed genes. **(D)** Confidence interval under lambda. **(E)** Eight hub genes, including three risk genes and five protective genes, were obtained.

A sum of 344 differentially expressed genes were identified between Clust1 and Clust2 (**Figure 8A**). Next, univariate Cox survival analysis determined 137 genes associated with prognosis (**Figure 8B**). The LASSO–Cox regression module was used to build a prognostic signature based on the expression matrix of the 137 genes. Subsequently, we identified an eight-gene signature module according to the optimal λ value (**Figures 8C, D**). RiskScore of HCC patients based on eight genes (**Figure 8E**) was calculated using the following formula:


RiskScore=0.011*RACGAP1−0.024*HAO2−0.055*OGDHL+0.122*ZWINT−0.069*CFHR3−0

044*CYP2C9+0.07*SFN−0.005*SPP2

### Validation of the prognostic model

**Figure 9:**
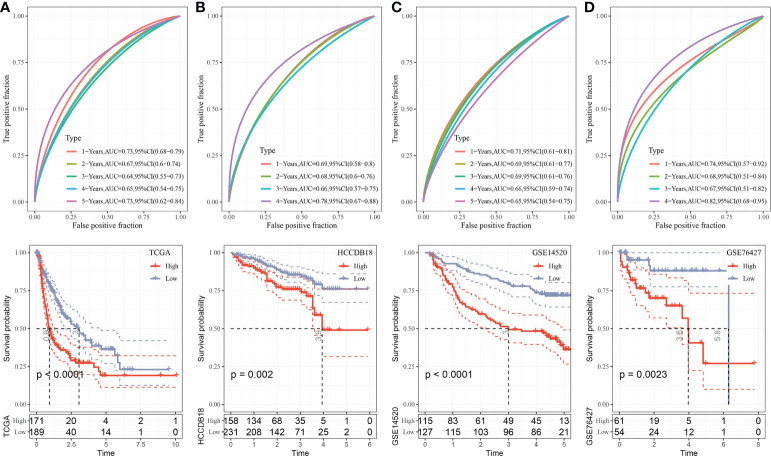
Validation of RiskScore. **(A)** ROC and K–M survival analysis of RiskScore in TCGA-LIHC dataset. **(B)** ROC and K–M survival analysis of RiskScore in the HCCDB18 dataset. **(C)** ROC and K–M survival analysis of RiskScore in the GSE14520 dataset. **(D)** ROC and K–M survival analysis of RiskScore in the GSE76427 dataset.

**Figure 10:**
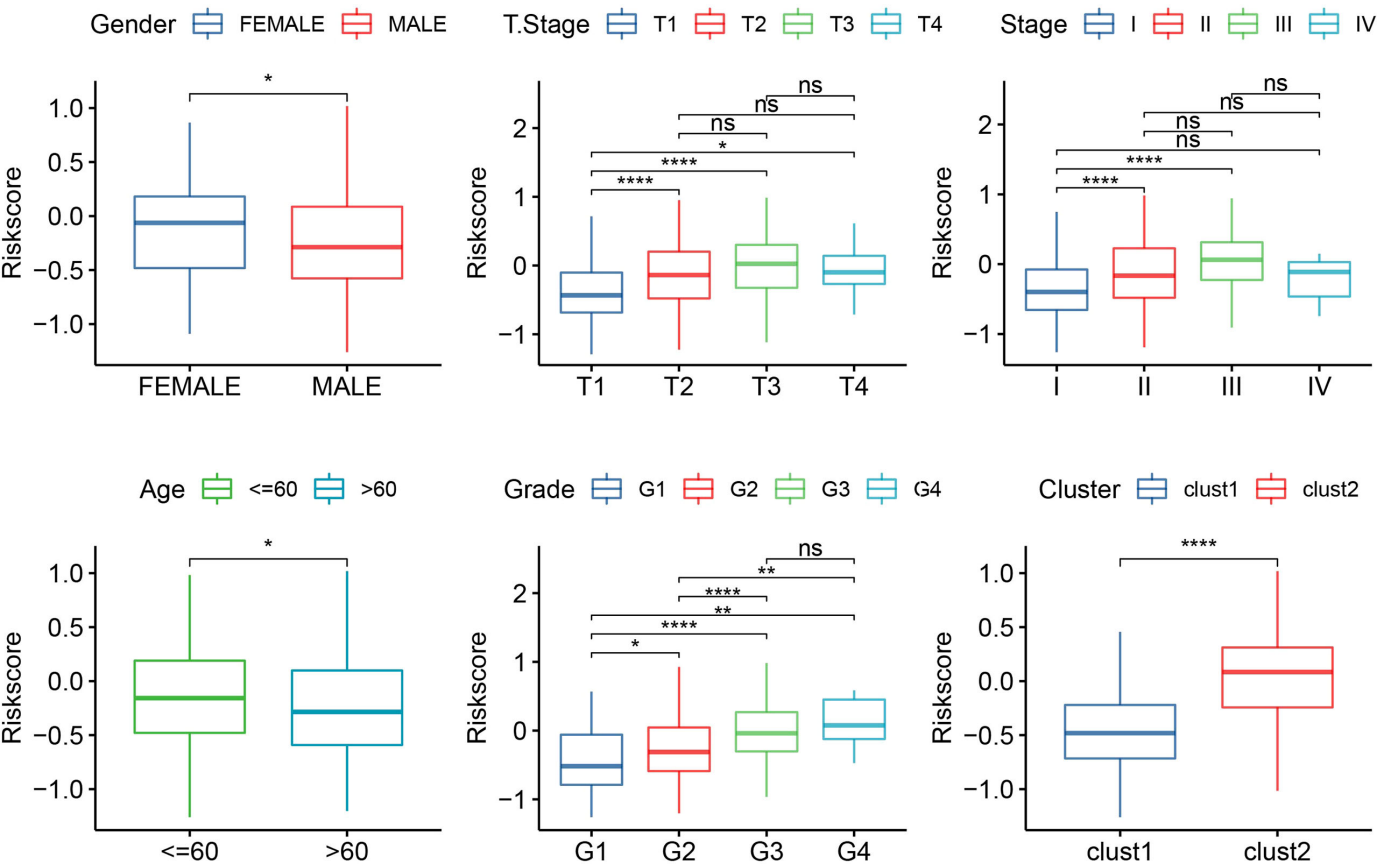
The RiskScore analysis in clinical features, including gender, T stage, stage, grade, age, and clusters. *p<0.05, **p<0.01, ****p<0.0001, ns, no significance.

The median RiskScore was the cutoff in classifying the samples into low-risk (RiskScore< median) and high-risk (RiskScore > median) groups. ROC and survival analyses were performed in TCGA-LIHC (**Figure 9A**), HCCDB18 (**Figure 9B**), GSE14520 (**Figure 9C**), and GSE76427 datasets (**Figure 9D**). The results revealed that the accuracy of the model was higher in predicting the 1‐, 2-, 3‐, 4-, and 5‐year survival rates in the above datasets, as all values of the area under the curve (AUC) were greater than 0.6. Results of the Kaplan–Meier survival analysis showed an overall survival higher in the low-risk group than the high-risk group. Female patients, those with a clinically advanced stage, younger samples (<=60), and clust2 showed a higher RiskScore (**Figure 10**).

### Analysis of immune infiltration and immunotherapy

**Figure 11:**
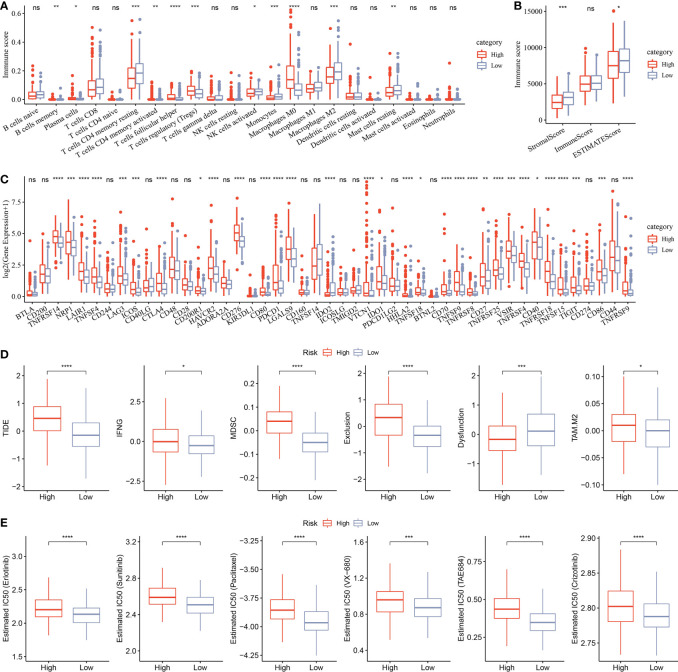
Analysis of immune infiltration. **(A)** Analysis of 22 immune cells using CIBERSORT. **(B)** Analysis of immune infiltration using ESTIMATE. **(C)** The expression levels of 42 immune check genes between low group and high group. **(D)** The differences of TIDE, IFNG, MDSC, Exclusion, Dysfunction, and TAM.M2 between low group and high group. **(E)** IC50 of traditional drugs in the low group and high group. *p<0.05, **p<0.01, ***p<0.001, ****p<0.0001, ns, no significance.

CIBERSORT analysis indicated that 11 of 22 immune cells were significantly higher in the low group that those in the high group (**Figure 11A**). ESTIMATE analysis showed that the low group had higher StromalScore and ESTIMATEScore (**Figure 11B**). Moreover, 31 immune checkpoint genes had obviously high expressions in the high group than in the low group (**Figure 11C**). Moreover, TIDE, IFNG, MDSC, Exclusion, and TAM.M2 were lower in the low group than in the high group, but Dysfunction was higher in the low group (**Figure 11D**), suggesting that the low group was more likely to benefit from immunotherapy. IC50 of erlotinib, sunitinib, paclitaxel, VX-680, TAE684, and crizotinib were higher in the high group, suggesting that patients in the high group were more sensitive to those drugs (**Figure 11E**).

### Nomogram

**Figure 12:**
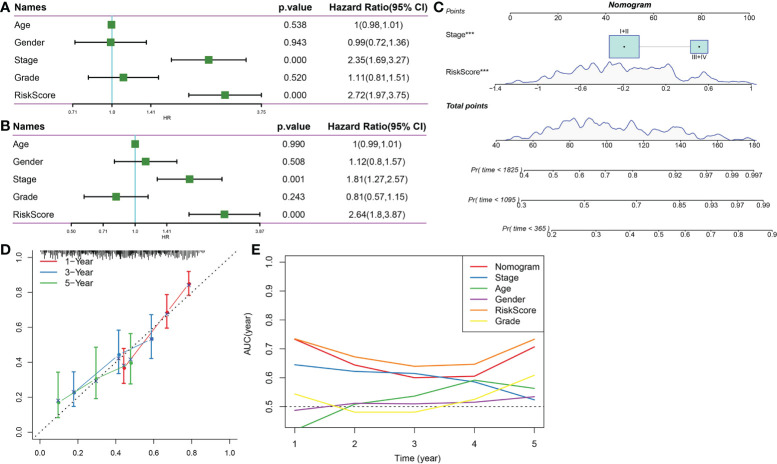
Nomogram. **(A)** Univariate Cox survival analysis. **(B)** Multivariate Cox survival analysis. **(C)** Construction of nomogram based on Stage and RiskScore. **(D)** The calibration curve proved that the prognostic nomogram was reliable and accurate. **(E)** AUC analysis of nomogram, RiskScore, age, stage, gender, and grade. ***p<0.001.

Univariate and multivariate Cox analyses indicated that Stage and RiskScore were independent prognostic factors (**Figures 12A, B**). Next, we constructed a prognostic nomogram based on Stage and RiskScore to predict the 1-, 3-, and 5-year overall survival of HCC patients (**Figure 12C**). The calibration curve proved that the prognostic nomogram was reliable and accurate (**Figure 12D**). The results of the AUC indicated that among other clinical variables, the RiskScore and Nomogram served as accurate prognostic indicators in clinical decision-making process (**Figure 12E**).

## Discussion

Although there are several methods to treat HCC, the effectiveness of treatment is limited by a late diagnosis at an advanced stage, which is usually accompanied by a high rate of disease recurrence (31). Therefore, identifying patients with high risk is crucial for doctors to determine the use of aggressive treatments. The TNM staging system developed by AJCC is a standard system for evaluating the prognosis of HCC patients (32, 33). However, clinical outcomes of patients with the same TNM stage could be greatly different sometimes. The current study investigated the prognostic significance of the autophagy score and developed a new nomogram model for predicting the OS of HCC patients. The autophagy score as an independent prognostic factor plays a key role in HCC survival. After internal verification, we found that nomogram prediction results were better than the TNM staging system.

Targeting autophagy is a new strategy in cancer immunotherapy (34). A study has shown that autophagy is associated with adaptive and innate immune responses and that it could be induced by immune receptors such as nucleotide oligomerization domain-like receptors (NLRs) and Toll-like receptors (35). Autophagy is involved in the lymphocyte development and antigen presentation, making autophagy a potential target in improving cancer immunotherapy (36). It has been further demonstrated that autophagy could facilitate cancer cells to effectively evade immune responses and immune surveillance. Thus, to prevent immune escape of cancer cells, targeting autophagy has gradually become a novel immunotherapeutic strategy (34). In this study, the autophagy score was developed to predict the response to immunotherapy, which further suggested that the autophagy score may be used to differentiate clinical patients and select more suitable treatment options.

A study reported a three-gene signature for predicting the survival outcome of HCC using scRNA and bulk RNA (37). Single-cell sequencing identifies three hub genes in HCC (38). However, in this study, we combined scRNA-seq and bulk-RNA to analyze HCC. The RiskScore model had eight genes, namely, HAO2, RACGAP1, OGDHL, ZWINT, CFHR3, CYP2C9, SFN, and SPP2. In human HCC tissues, several studies reported an obviously lower HAO2 expression, which was associated with a worse HCC prognosis (39); moreover, HAO2 was overexpressed and HCC cell invasion, migration, and proliferation were inhibited (40, 41). RACGAP1 is frequently overexpressed and associated with shorter survival time of HCC patients (42, 43). Silencing OGDHL would induce lipogenesis and affect sorafenib’s chemosensitization effect on HCC cells (44). ZWINT upregulation showed a significant association with unfavorable survivals and clinicopathological features of HCC patients (45). CFHR3 overexpression was correlated with a favorable prognosis for HCC patients (46). CYP2C9 is involved in the metabolism of many carcinogens and drugs and is downregulated in HCC (47). SFN significantly inhibited the proliferation of HepG2 cells (48). So far, there was no study discussing the potential functions of SPP2 in HCC, but results indicated that SPP2 was associated with the stability of spliceosome and chromatin (49). Hence, we speculated that SPP2 dysregulation may lead to carcinogenesis and disease progression.

Some limitations existed in this study. Firstly, the sample lacked clinical follow-up information; thus, factors such as the presence of other health conditions were not considered during the identification of the biomarkers. Secondly, the results obtained by bioinformatics analysis alone were not convincing enough, which requires further experimental verification. In addition, the molecular processes and signaling pathways obtained from TCGA cases alone are not sufficient and should be confirmed by further studies.

In conclusion, we constructed a prognostic prediction model which consisted of HAO2, RACGAP1, OGDHL, ZWINT, CFHR3, CYP2C9, SFN, and SPP2 for HCC, which provided new ideas for the prognostic treatment of HCC patients. However, the main limitation of our results was that our study was conducted in a public database, and further experiments are needed for in-depth study.

## Data availability statement

The original contributions presented in the study are included in the article/**Supplementary Material**. Further inquiries can be directed to the corresponding authors.

## Author contributions

XL and JL conducted the statistical analyses of the data and prepared the draft manuscript. QW and LB edited the manuscript. JX, XH, SL, and QL provided critical comments to the manuscript. All authors contributed to the article and approved the submitted version.

## Funding

The present study was supported by the Henan Medical Science and Technology Key Project: The Effect of Hyperammonemia on Hepatocyte Regeneration and Metabolism After Liver Failure and Its Clinical Treatment (20170219).

## Conflict of interest

Author SL was employed by Hangzhou Mugu Technology Co., Ltd.

The remaining authors declare that the research was conducted in the absence of any commercial or financial relationships that could be construed as a potential conflict of interest.

## Publisher’s note

All claims expressed in this article are solely those of the authors and do not necessarily represent those of their affiliated organizations, or those of the publisher, the editors and the reviewers. Any product that may be evaluated in this article, or claim that may be made by its manufacturer, is not guaranteed or endorsed by the publisher.
