# Supplementary material for: Analysis on heterogeneity of hepatocellular carcinoma immune cells and a molecular risk model by integration of scRNA-seq and bulk RNA-seq
Source: Front Immunol. 2022 Oct 13;13:1012303. doi: 10.3389/fimmu.2022.1012303 (PMC9606610; doi:10.3389/fimmu.2022.1012303)
Supplement: Supplementary file 1 [file DataSheet_1.docx]

**Supplementary figure legend**

**Figure S1**

Workflow

**Figure S2**

Clustering and dimension reduction analysis of single cell data. A: Correlation analysis of UMI and number of mRNA, Mitochondrial gene. B: MRNA /UMI/ mitochondrial content /rRNA content of samples before filtration. C: MRNA /UMI/ mitochondrial content /rRNA content of samples after filtration. D: The 10 sample distribution of PCA dimension reduction and the anchor point diagram of PCA.

**Figure S3**

Cell annotation of 11 clusters was performed by classical markers of immune cells.

**Figure S4**

The analysis of 5 the autophagy pathways scores in Grade1 to Grade4.
