# Supplementary figures and images for: Analysis on heterogeneity of hepatocellular carcinoma immune cells and a molecular risk model by integration of scRNA-seq and bulk RNA-seq
Source: Front Immunol. 2022 Oct 13;13:1012303. doi: 10.3389/fimmu.2022.1012303 (PMC9606610; doi:10.3389/fimmu.2022.1012303)

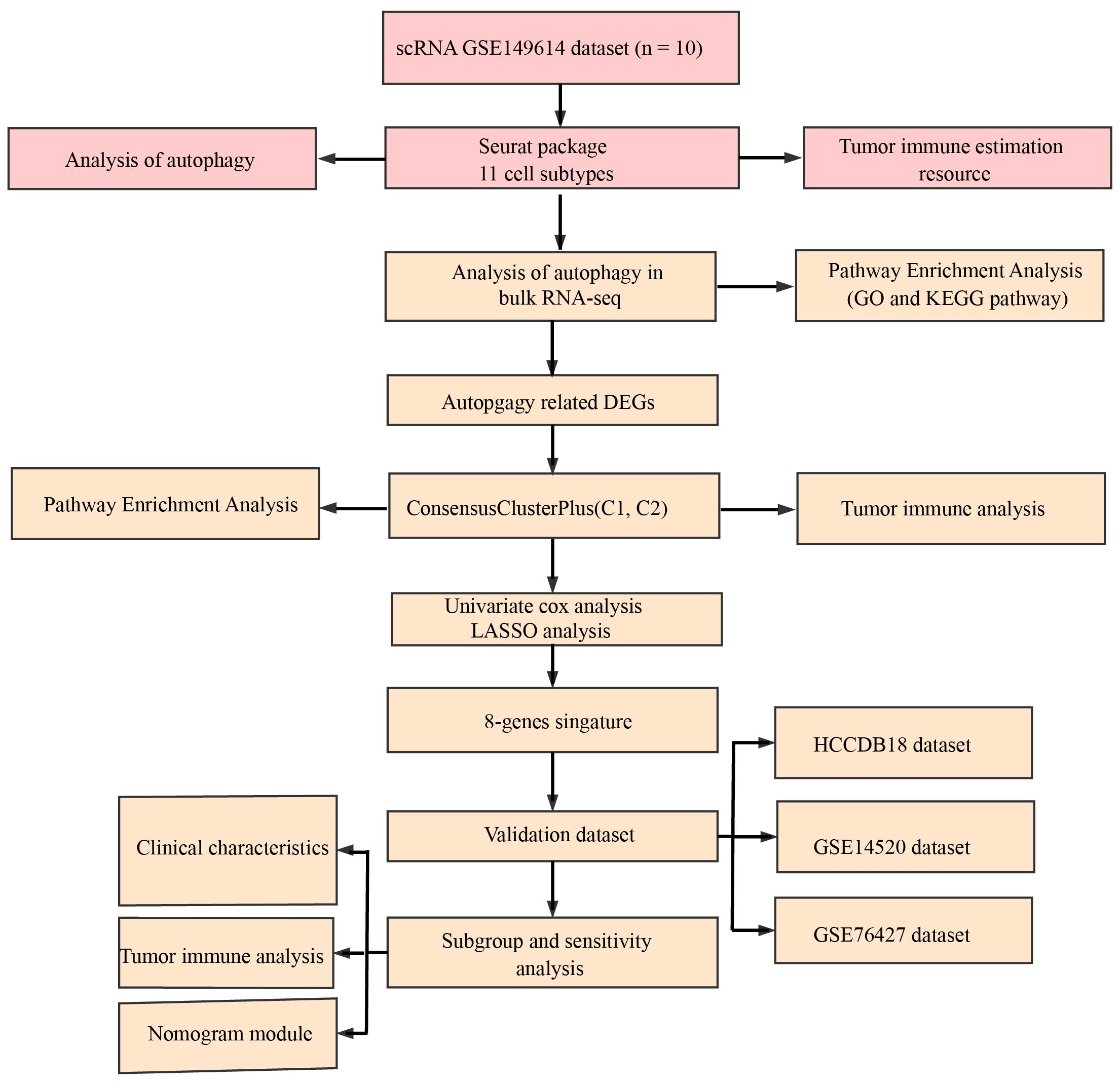

Supplement: Supplementary file 2 [file Image_1.jpeg]

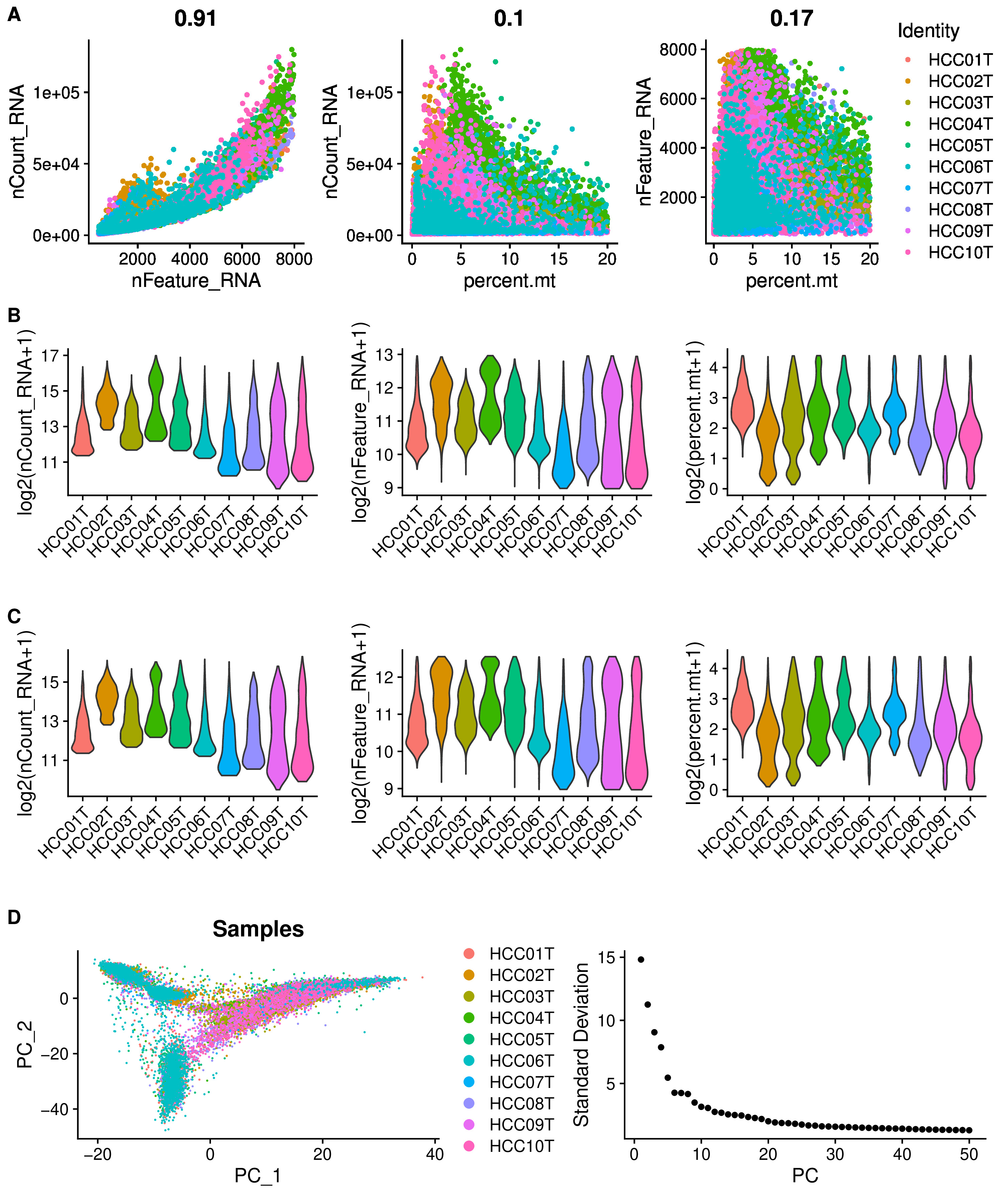

Supplement: Supplementary file 3 [file Image_2.jpeg]

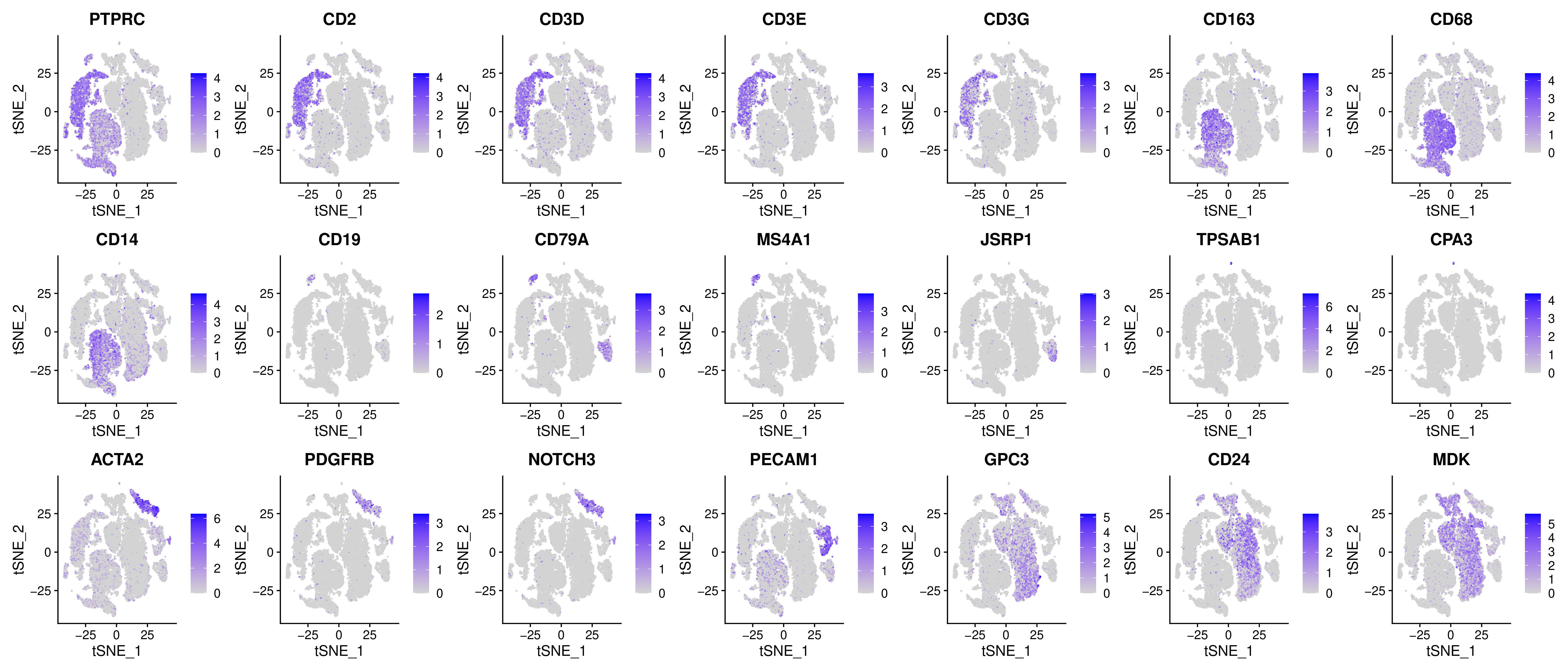

Supplement: Supplementary file 4 [file Image_3.jpeg]

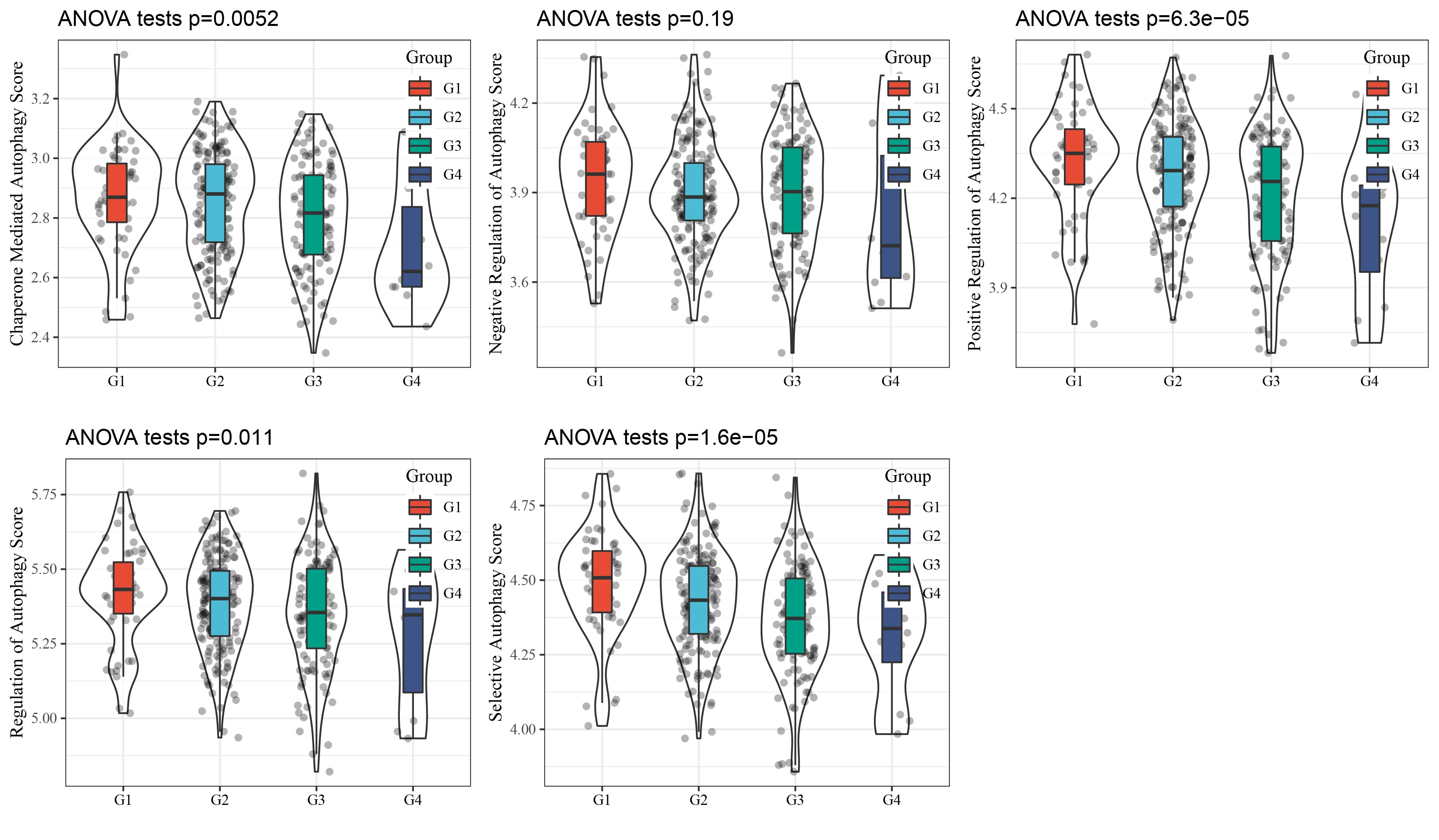

Supplement: Supplementary file 5 [file Image_4.jpeg]
